# Simultaneous Determination of Six Active Components in* Oviductus Ranae* via Quantitative Analysis of Multicomponents by Single Marker

**DOI:** 10.1155/2017/9194847

**Published:** 2017-10-24

**Authors:** Shihan Wang, Yang Xu, Yanwei Wang, Huailei Yang, Zuying Lv, Xiangqun Jin, Yongsheng Wang

**Affiliations:** ^1^School of Pharmacy, Jilin University, Changchun, Jilin 130021, China; ^2^Department of Pharmaceutics, Changchun Medical College, Changchun, Jilin 130021, China; ^3^Chinese Institute of Jilin Ginseng, Changchun, Jilin 130021, China

## Abstract

A method, quantitative analysis of multicomponents by single marker (QAMS), was established in this article to investigate the quality control of a traditional Chinese medicine,* Oviductus Ranae*. 7-Hydroxycholesterol, 7-ketocholesterol, 4-cholesten-3-one, stigmasterol, 7-dehydrocholesterol, and cholesterol were selected as the indexes of quality evaluation of* Oviductus Rana.* The determination was achieved on an Agilent HC-C18 column (4.6 mm × 250 mm, 5 *μ*m) using methanol with water (87 : 13 v/v) as mobile phase at the flow rate of 2.0 mL/min. Cholesterol was used as an internal standard to determine the relative correction factors between cholesterol and other steroidal constituents in* Oviductus Ranae*. The contents of those steroidal constituents were calculated at the same time. To evaluate the QAMS method, an external standard method was used to determine the contents of six steroidal constituents. No significant difference was observed when comparing the quantitative results of QAMS with the results of external standard method. The proposed QAMS method was proved to be accurate and feasible based on methodological experiments. QAMS provided a simple, efficient, economical, and accurate way to control the quality of* Oviductus Ranae*.

## 1. Introduction

*Oviductus Ranae*, a traditional Chinese medicine (TCM), can be obtained from dry oviduct of female* Rana temporaria chensinensis *David [1]. It has increasingly attracted worldwide attention because of its effect on nourishing Yin and moistening lung, strengthening the spleen and stomach, nourishing the kidney, and strengthening the essence as well. Pharmacology study showed it also has curative effects such as antitussive, antiasthmatic, antiaging, and antifatigue effects and enhancing the efficacy of immunity [2–5]. The challenge preventing its clinical practice is the uncontrollable quality of* Oviductus Ranae.* Determination method for the components of* Oviductus Ranae* has not been introduced in* Chinese Pharmacopoeia* (Chp; 2015 edition). Current method for the quality control of* Oviductus Ranae *involves using single component or a marker compound as the internal standard substance [6–8]. However, high-quality standard compounds or references are quite expensive and relying on single component of quality control is insufficient. Therefore, establishing a low-cost, reliable, and efficient approach is significantly important.

**Figure 1:**
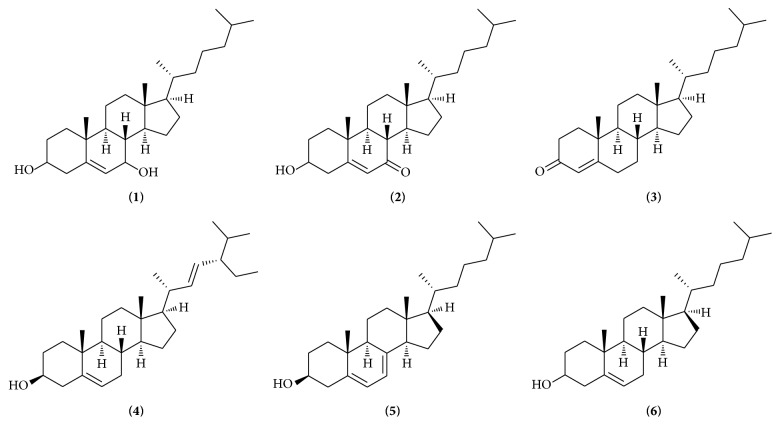
The chemical structures of six analytes: 7-hydroxycholesterol (**1**), 7-ketocholesterol (**2**), 4-cholesten-3-one (**3**), stigmasterol (**4**), 7-dehydrocholesterol (**5**), and cholesterol (**6**).

Quantitative analysis of multicomponents by single marker (QAMS) has been regarded as an alternative to the quality control of TCMs [9–15]. The relative correction factor (RCF) of contents in* Oviductus Ranae* is calculated using the reference substance of each analyte [16–18]. The RCF of the other six analytes were determined. Their contents were calculated based on RCF. Steroidal constituents in* Oviductus Ranae* have significant pharmacological activities which are mainly exhibited on the aspects of antifatigue, immunity enhancement, antiaging, maintaining the balance of hormones. In addition, some steroids can be transformed into other steroid hormones with good activities through biological transformation [19–33]. To develop a QAMS method for the simultaneous determination of six active components in* Oviductus Ranae*, cholesterol was chosen as the internal standard substance. Six active steroidal components were selected as the indexes of quality evaluation of* Oviductus Ranae *(Figure 1).

## 2. Experimental

### 2.1. Instrument and Chromatographic Conditions

Chromatographic separation was achieved on Agilent 1260 high performance liquid chromatography (HPLC) equipped with DAD detector (G1315B), automatic sampling device (G1329B), Agilent Chemstation workstation, and Waters Alliance E2695. Another high performance liquid chromatography was equipped with 2489 UV detector, automatic sampling device (E2695), and Empower workstation. Agilent HC-C18, Amethyst C18-P, and Eclipse XDB-C18 (250 mm × 4.6 mm, 5 *μ*m) were employed during the experiment under the following chromatographic conditions: the mobile phase was composed of methanol and water (87 : 13 v/v) under the detection wavelength of 205 nm, 240 nm, and 280 nm at the flow rate of 2.0 mL·min^−1^. The column temperature was maintained at 35°C, and the injection volume was 10 *μ*L. KQ-400KDB high power digital ultrasonic cleaner was purchased from Kunshan Ultrasonic Instrument Co. Ltd. RE-52-99 rotary evaporator was purchased from Shanghai Ya Rong Biochemical Instrument Factory. WP-UP-III-10 ultrapure water was purchased from Sichuan Water Treatment Equipment Co. Ltd.

### 2.2. Reagents and Materials

Cholesterol (batch number: 111618-200301) was purchased from China Food and Drug Verification Research Institute (Beijing, China). 4-Cholesten-3-one (batch number: S45539-479) was purchased from American sigma company (USA). 7-Dehydrogenation cholesterol (batch number: CDCT-12115000) was purchsed from Dr. Ehrenstorfer (Germany). Stigmasterol (batch number: A0510) was purchased from Shanghai Yao Yun Biological Science and Technology Co., Ltd. 7-Ketocholesterol and 7-hydroxycholesterol were prepared by laboratory according to previous reports (content: more than or equal to 98%) [34]. Fourteen batches of* Oviductus Ranae* were collected from different regions in China, which were conducted by Professor Dacheng Jiang from Changchun University of Traditional Chinese Medicine and proved to be dry oviduct of female* Rana temporaria chensinensis *David. The details were showed in Table 1. Methanol (Fisher, America) was of chromatographic grade, and other reagents (Beijing Chemical Industry Factory) were of analytical grade. Ultrapure water was prepared by water instrument WP-UP-III-10.

### 2.3. Preparation of Mixed Standard Solution

Substances of 7-hydrocholesteol, 7-ketocholesterol, 4-cholesten-3-one, 7-dehydrogenation cholesterol, cholesterol, and stigmasterol were weighed precisely and dissolved into methanol to prepare standard solutions with the concentration of 1.579 × 10^2^ *μ*g/mL, 32.525 *μ*g/mL, 20.225 *μ*g/mL, 9.775 *μ*g/mL, 4.00 × 10^3^ *μ*g/mL, and 25.738 *μ*g/mL, respectively. The standard solutions were stored at 4°C in refrigerator.

### 2.4. Preparation of Test Solutions

About 2.0 g* Oviductus Ranae* powder (40-mesh sieve) was accurately weighed and placed in a Erlenmeyer flask. 40 mL dichloromethane was added to the flask and extracted for 20 min via ultrasonic method with the power of 400 W and the frequency of 40 KHz. The extraction was repeated for three times and filtered. The combined filtrates were concentrated via roller evaporator. The residue was dissolved into 2 mL of methanol in volumetric flask. The solution was then filtrated through a 0.22 *μ*m membrane. The filtrate was regarded as the test solution.

### 2.5. Method Validation

#### 2.5.1. System Adaptability Test

**Figure 2:**
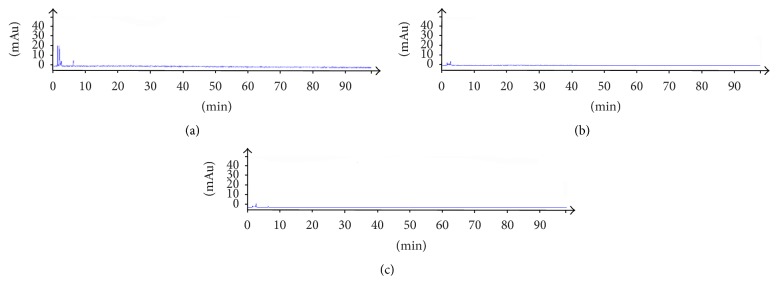
Blank solvent of three detection wavelengths including (a) 205 nm, (b) 240 nm, and (c) 280 nm.

**Figure 3:**
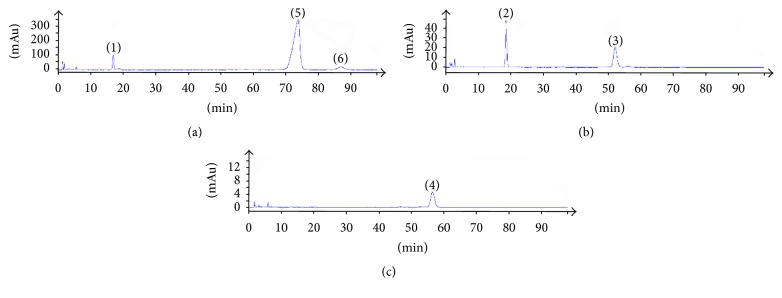
The chromatograms of mixed reference substance ((1) 7-hydroxycholesterol; (2) 7-ketocholesterol; (3) cholest-4-en-3-one; (4) 7-dehydrocholesterol; (5) cholesterol; (6) stigmasterol) at three detection wavelengths ((a) 205 nm; (b) 240 nm; (c) 280 nm).

**Figure 4:**
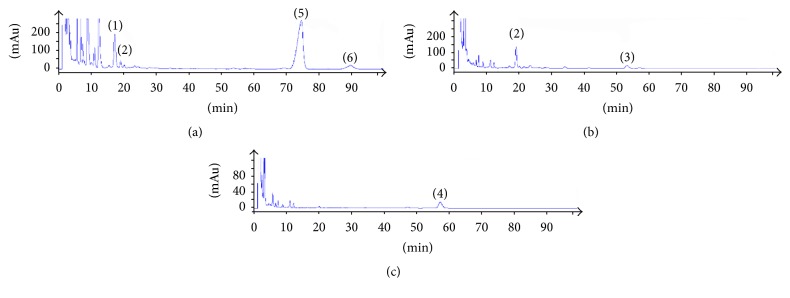
The chromatograms of test samples ((1) 7-hydroxycholesterol; (2) 7-ketocholesterol; (3) cholest-4-en-3-one; (4) 7-dehydrocholesterol; (5) cholesterol; (6) stigmasterol) at three detection wavelengths ((a) 205 nm; (b) 240 nm; (c) 280 nm).

Blank solvent, standard solution, and sample solution of each substance were analyzed based on the chromatographic condition of Section 2.1 and the results showed in Figures 2, 3, and 4.

#### 2.5.2. Liner Range

A series volume of mixed standard solutions was transferred by volume with 5 *μ*L, 10 *μ*L, 15 *μ*L, 20 *μ*L, 30 *μ*L, 40 *μ*L, 50 *μ*L, and 60 *μ*L and injected into HPLC, respectively. Chromatogram was recorded and the standard curve was drawn with peak area (*Y*) as the vertical coordinate and the content of the measured substance (*X*) as horizontal coordinate. The mixed control solutions were diluted with methanol. The limits of detection (LOD) and the limits of quantification (LOQ) were determined by three times and ten times of the signal noise ratio, respectively.

#### 2.5.3. Precision

An intraday precision test was carried out by the following method. The same mixed control solution was transferred in the same day for six times in a row. RSD% was calculated according to the chromatographic peak area of the components.

#### 2.5.4. Stability Test

The sample solution was injected into HPLC at 0, 2, 4, 6, 8, 10, 12, and 24 h after being prepared and peak area of each component was recorded and RSD% was calculated.

#### 2.5.5. Repeatability Test

The same batch of* Oviductus Ranae* drug powder was transferred to prepare six sample solutions in parallel according to method of Section 2.4. The chromatographic peak area of each component was recorded. The content and RSD% were calculated.

#### 2.5.6. Recovery Test

About 1.0 g of the same batch of* Oviductus Ranae* powder was weighed precisely, and then a certain amount of control sample solution was added to the sample according to the proportion of sample content-reference substance (1 : 1) to prepare six sample solutions in parallel referring to method of Section 2.3. The six sample solutions were injected into HPLC, respectively, and the peak areas were recorded to calculate average recovery rate and RSD% of each sample.

#### 2.5.7. Durability Test

The durability test was conducted by two different instruments which were Agilent 1260 and Waters E2695. Three different chromatographic columns were applied including Agilent HC-C18, Eclipse XDB-C18, and Amethyst C18-P (250 mm × 4.6 mm, 5 *μ*m). Analysis was determined at the flow rate of 2.2 mL·min^−1^, 2.0 mL·min^−1^, and 1.8 mL·min^−1^ with the column temperature of 30°C, 35°C, and 40°C. The content and RSD% of the six steroid components in the same batch were calculated. The separation degree of the charmatographic column was investigated. The number of theoretical plates was also investigated

## 3. Results and Discussion

### 3.1. Method Validation

#### 3.1.1. System Adaptability Test

The theoretical plates were selected according to the separation resolution between the analytes and impurities. After several tests of standards and testing samples, we found when theoretical plates are bigger than or equal to 3500 the analytes have good resolution in chromatographic peaks. The results of system adaptability are showed in Figures 2, 3, and 4. The resolutions of 7-hydrocholesteol, 7-ketocholesterol, 4-cholesten-3-one, 7-dehydrogenation cholesterol, cholesterol, and stigmasterol were more than 1.5 and the number of theoretical plates was greater than 3500.

#### 3.1.2. Liner Range

The results were showed in Table 2. High coefficient of determination values (*R*^2^ > 0.9997) showed good linearity. LOD and LOQ of six substances were within the range of 0.055–0.100 *μ*g/mL and 2.445–1.001 × 10^3^ *μ*g/mL, which showed a high sensitivity under the established chromatographic condition.

#### 3.1.3. Precision

The results showed that RSD% values of the peak area of 7-hydrocholesteol, 7-ketocholesterol, 4-cholesten-3-one, 7-dehydrogenation cholesterol, cholesterol, and stigmasterol were 0.5%, 0.8%, 0.8%, 1.1%, 0.3%, and 0.5%, which showed a good instrument precision.

#### 3.1.4. Stability Test

The results showed that RSD% values of the peak area of 7-hydrocholesteol, 7-ketocholesterol, 4-cholesten-3-one, 7-dehydrogenation cholesterol, cholesterol, and stigmasterol were 0.9%, 0.7%, 1.1%, 1.0%, 0.1%, and 0.4% (RSD% ≤ 2.0%), indicating that the tested sample solution was stable within 24 h.

#### 3.1.5. Repeatability Test

The results revealed that average mass fraction and RSD% values of the peak area of 7-hydrocholesteol, 7-ketocholesterol, 4-cholesten-3-one, 7-dehydrogenation cholesterol, cholesterol, and stigmasterol were 74.30 *μ*g/mL, 17.70 *μ*g/mL, 7.29 *μ*g/mL, 5.17 *μ*g/mL, 3.48 *μ*g/mL, and 15.13 *μ*g/mL and 0.6%, 0.4%, 0.5%, 0.9%, 1.0%, and 0.8%. The method had good repeatability.

#### 3.1.6. Recovery Test

The results showed that average recovery rate and RSD% of the peak area of 7-hydrocholesteol, 7-ketocholesterol, 4-cholesten-3-one, 7-dehydrogenation cholesterol, cholesterol, and stigmasterol were 98.0%, 98.1%, 99.1% 99.2%, 98.3%, 98.9%, and 1.3%, 1.5%, 1.6%, 1.0%, 0.9%, and 1.5%, illustrating that the method was of good accuracy.

#### 3.1.7. Durability Test

The RSD% results of mass fractions, determined in all the above conditions, were less than 3.1%, which showed separation effect was good. According to the tests, the determination conditions of six steroidal components in* Oviductus Ranae* were wide and the method exhibited good durability.

### 3.2. Quantitative Analysis of Multicomponents by Single Marker

#### 3.2.1. Calculation of Relative Correction Factor

In standard curve *Y* = *a* × *X* + *b*, *X* = (*Y* − *b*)/*a* = *Y*/*a* − *b*/*a*, and value of *b*/*a* can be ignored when the value of *a*/*b* is greater than 100 because the value of *b* is usually caused by error. At this point, the formula can be directly calculated as *X* = *Y*/*a*. Therefore, the relative correction factor can be calculated by the ratio of the slope (*a*). The relative correction factor calculation formula is shown as follows:

(1) $$\begin{array}{c} f_{K/S}=\frac{a_{K}}{a_{S}}, \end{array}$$

where *a*_*S*_ is the slope of reference and *a*_*K*_ is the slope of the other component to be measured [33]. Cholesterol was selected as an internal reference (1.000) for the quantitative analysis of other five steroids, including 7-hydrocholesteol, 7-ketocholesterol, 4-cholesten-3-one, 7-dehydrogenation cholesterol, and stigmasterol with the detection wavelengths of 205 nm, 240 nm, and 280 nm, respectively. The relative correction factors of 7-hydrocholesteol and stigmasterol were 1.5354 and 8.2591 under 205 nm. The relative correction factors of 7-ketocholesterol and 4-cholesten-3-one were 5.3021 and 5.4305 under 240 nm. The relative correction factor of 7-dehydrogenation cholesterol was 3.0043.

#### 3.2.2. Reproducibility of RCF

In this paper, the effects of two sets of chromatographic systems (Agilent 1260 and Waters E2695), three kinds of chromatographic columns (Agilent HC-C18, Eclipse XDB-C18, and Amethyst C18-P (250 mm × 4.6 mm, 5 *μ*m), and different column temperatures and different flow rates on RCF were investigated. Results showed RSDs measured in different conditions were all less than 1.19%. The experiments were repeated by different researchers in different laboratories with the same conditions, and all results showed RSDs were less than 2.0%. indicating the RCF calculated by the established method has good reproducibility.

#### 3.2.3. Location of Chromatographic Peak of the Measured Component

**Figure 5:**
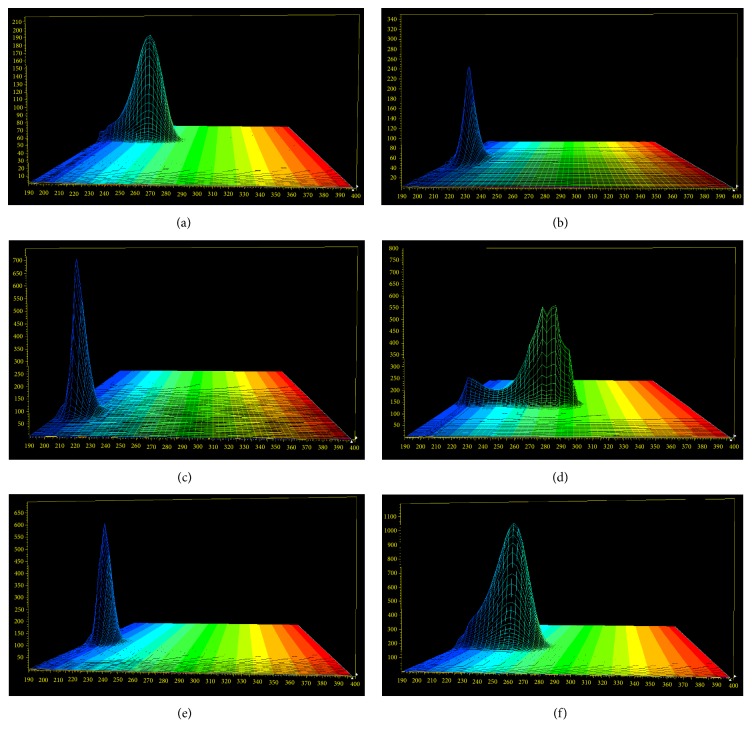
(a) Cholest-4-en-3-one, (b) stigmasterol, (c) cholesterol, (e) 7-hydroxycholesterol, and (f) 7-ketocholesterol. 3D absorption spectroscopy of each component.

The relative retention time was achieved based on the ratio of retention time of measured component (*k*) and internal standard (*s*). The formula was shown as follows:

(2) $$\begin{array}{c} Rt_{R}=\frac{t_{Rk}}{t_{Rs}}, \end{array}$$

where *k* is the component to be measured and *s* is regarded as internal reference cholesterol. The relative retention time between internal reference cholesterol and other components was calculated through formula (2). The effects of different chromatographic systems, column temperatures, and flow rates on RSDs of relative retention time were evaluated. Results showed RSDs of relative retention time between tested components and internal standard cholesterol were less than 0.67% and the RSD% results of durability study were also less than 4.91%. On the other hand, each tested component was scanned via the diode array detector (DAD). 3D absorption spectrum of each compound was obtained, showed in Figure 5. Peak time of each component can be predicted through the relative retention time, but if other peaks existed near the chromatographic peaks, chromatographic peaks can also be located by overall shape or 3D absorption spectra. Therefore, chromatographic peak can be accurately located in a certain extent by the method established above.

#### 3.2.4. Comparison of the Results of QAMS Method and External Standard Method

Fourteen batches of* Oviductus Ranae* powder purchased from different regions were prepared based on the method of Section 2.4. Samples obtained from each region were prepared for two independent copies in parallel and injected into HPLC based on the method of Section 2.1. Subsequently, chromatographic peak area of each tested component was recorded. Then, the content of each component was calculated by the method of ES and QAMS, respectively. Results were shown in Table 3. There were no significant differences between the results of two methods. In addition, the relative errors were less than 2%, which indicated that the method established above was accurate and reliable.

### 3.3. Discussion

Cholesterol was selected as the internal reference substance of QAMS method, because cholesterol is one of the main components of* Oviductus Ranae* with high amount, low price, and easy acquisition. Furthermore, there were no significant differences between the content determination results of QAMS method and ES method when cholesterol was regarded as internal reference material, and the relative error was less than 2% as well.

In this paper, to obtain a good separation degree, higher theoretical plate number, and good symmetry of chromatographic peak, different mobile phase conditions were investigated for system adaptability test. It was found that the chromatographic peak of all the tested components could achieve baseline separation with the adjacent peaks using methanol and water (87 : 13 v/v) as mobile phase. To confirm the best preparation method of the tested sample solution, different extraction solvent (methanol, dichloromethane, and chloroform), extraction method (cold soak, ultrasonic, and reflux), amount of the solvent (30 mL, 40 mL, and 50 mL), and extraction time (10 min, 20 min, and 30 min) were studied. The best preparation process was considered as extracting with 40 mL dichloromethane for 20 min via ultrasonic method based on the single factor investigation experiment mentioned above.

It was critical to select suitable detection wavelength for achieving a good separation. The selection of detection wavelength relied on the UV absorption characteristics of the internal reference material and the separation of other components in the test samples. Internal reference cholesterol, 7-hydroxycholesterol, and stigmasterol all exhibited good UV absorption and good separating degree at 205 nm. The wavelength of 205 nm was regarded as one of the detection wavelengths for the reason above. However, detection of cholest-4-en-3-one and 7-dehydrocholesterol was not sensitive at 205 nm because of less content in* Oviductus Ranae*. Thus, based on the characteristics of UV absorption of these two substances, 240 nm and 280 nm were selected as the detection wavelengths, respectively. The detection wavelength of cholesterol, 7-hydroxycholesterol, and stigmasterol was 205 nm. The detection wavelength of cholest-4-en-3-one and 7-ketocholesterol was determined as 240 nm, and 7-dehydrocholesterol was detected at 280 nm.

In this experiment, two kinds of method were investigated including the difference of retention time and relative retention time for locating chromatographic peaks when using cholesterol as internal reference substance. Compared with the results of retention time difference method, the relative retention time was relatively stable, and the RSD% was less than 4.9%. Thus, the chromatographic peak position can be located according to the relative retention time of chromatographic peaks between tested compounds and internal standard cholesterol, the chromatograms of the overall shape, and the 3D absorption spectra information of six components in* Oviductus Ranae*.

## 4. Conclusion

In this paper, HPLC-DAD method was adopted for determination of relative retention time and relative correction factor of six steroids in* Oviductus Ranae* for the first time. A QAMS method was developed for determining the contents of six kinds of steroidal constituents simultaneously. The QAMS method established in this study has been proved by method validation methodology to be accurate, feasible, and reliable for determination of contents of six steroidal components and can be applied to the quality control of* Oviductus Ranae*.

## Figures and Tables

**Table 1:** Batch and origin of *Oviductus Ranae*.

NO.	Origin	Year	Batch
S1	Tonghua, Jilin Province	2014	201401
S2	Tonghua, Jilin Province	2014	201402
S3	Huadian, Jilin Province	2015	201503
S4	Huadian, Jilin Province	2015	201504
S5	Heihe, Heilongjiang Province	2015	201505
S6	Dunhua, Jilin Province	2015	201506
S7	Jingyu, Jilin Province	2015	201507
S8	Jingyu, Jilin Province	2015	201508
S9	Fangzheng, Heilongjiang Province	2015	201509
S10	Mudanjiang, Heilongjiang Province	2015	201510
S11	Antu, Jilin Province	2015	201511
S12	Dandong, Liaoning Province	2015	201512
S13	Hebei Province	2015	201513
S14	Hulin, Heilongjiang Province	2015	201514

**Table 2:** The linear equations, coefficient of determination, and linear range of six components.

Substance	Liner equation	*R* ^2^	Liner range/*μ*g/mL	LOD/*μ*g/mL	LOQ/*μ*g/mL
Cholesterol	*y* = 206.39*x* − 1.93	1.0000	1.00 × 10^3^–1.20 × 10^3^	0.075	1.001 × 10^3^
Stigmasterol	*y* = 1704.6*x* + 16.13	0.9997	6.43–77.21	0.085	6.435
7-Hydrocholesteol	*y* = 316.9*x* + 3.13	0.9998	39.47–473.70	0.060	39.475
7-Ketocholesterol	*y* = 1094.3*x* − 0.33	1.0000	8.13–97.57	0.065	8.130
4-Cholesten-3-one	*y* = 1120.8*x* + 1.10	1.0000	5.05–60.67	0.055	5.055
7-Dehydrogenation cholesterol	*y* = 620.05*x* − 0.42	0.9999	2.44–29.32	0.100	2.445

**Table 3:** Determination results and comparison of different method of six steroid components in *Oviductus Ranae*.

NO.	Method	Cholesterol	7-hydroxycholesterol		7-ketocholesterol		Cholest-4-en-3-one		Stigamasterol		7-dehydroxycholesterol	
		*μ*g·g^−1^	*μ*g·g^−1^	RSD%	*μ*g·g^−1^	RSD%	*μ*g·g^−1^	RSD%	*μ*g·g^−1^	RSD%	*μ*g·g^−1^	RSD%
(1)	ES	3.5 × 10^3^	75.1	0.1	17.6	−0.6	7.3	0.4	15.1	0.3	5.2	−0.8
	QAMS		75.2		17.5		7.3		15.1		5.1	
(2)	ES	3.8 × 10^3^	77.9	−0.1	19.4	−0.6	8.4	−0.4	15.4	0.3	5.5	0.7
	QAMS		77.8		19.3		8.4		15.5		5.5	
(3)	ES	2.3 × 10^3^	59.5	−0.1	13.1	0.6	6.6	−0.5	11.2	0.4	4.1	1.0
	QAMS		59.4		13.1		6.5		11.3		4.1	
(4)	ES	3.5 × 10^3^	70.2	0.1	16.9	0.7	7.4	0.7	14.9	−0.4	5.2	0.8
	QAMS		70.3		17.0		7.5		14.9		5.3	
(5)	ES	3.2 × 10^3^	66.1	−0.1	16.0	0.6	7.4	0.4	14.8	0.3	4.8	−0.8
	QAMS		66.0		16.1		7.4		14.8		4.7	
(6)	ES	3.0 × 10^3^	64.8	0.1	15.5	−0.6	7.1	0.4	14.6	0.3	4.2	1.0
	QAMS		64.8		15.4		7.1		14.6		4.3	
(7)	ES	3.1 × 10^3^	67.7	−0.2	15.9	−0.8	7.3	0.7	14.7	−0.3	4.5	0.9
	QAMS		67.6		15.8		7.3		14.6		4.6	
(8)	ES	3.2 × 10^3^	60.7	0.2	14.5	−0.8	6.9	0.4	14.4	0.4	4.9	−0.8
	QAMS		60.8		14.3		6.9		14.5		4.8	
(9)	ES	3.9 × 10^3^	81.2	0.1	22.1	0.6	9.2	0.6	15.6	−0.4	6.1	0.8
	QAMS		81.3		22.2		9.2		15.6		6.1	
(10)	ES	2.5 × 10^3^	60.1	−0.1	13.4	0.8	6.7	−0.6	12.7	−0.4	4.1	1.0
	QAMS		60.0		13.5		6.6		12.7		4.2	
(11)	ES	3.3 × 10^3^	72.4	−0.12	17.8	−0.8	7.3	−0.4	14.1	−0.4	4.6	0.9
	QAMS		72.3		17.7		7.3		14.1		4.7	
(12)	ES	3.6 × 10^3^	70.6	−0.1	18.4	0.7	7.6	0.5	14.3	0.3	5.2	0.8
	QAMS		70.5		18.5		7.7		14.3		5.3	
(13)	ES	3.9 × 10^3^	78.9	−0.1	21.2	0.7	8.7	−0.3	15.3	0.3	5.9	0.9
	QAMS		78.8		21.4		8.7		15.4		5.9	
(14)	ES	2.7 × 10^3^	61.5	0.1	12.3	−0.7	6.3	0.6	13.2	0.4	4.0	0.8
	QAMS		61.6		12.2		6.3		13.2		4.0	
(15)	ES	2.1 × 10^3^	53.4	−0.1	11.6	0.9	5.8	−0.5	11.8	0.3	3.7	1.1
	QAMS		53.3		11.7		5.8		11.8		3.8	
(16)	ES	2.7 × 10^3^	60.1	0.2	12.8	0.8	6.7	0.6	13.6	0.4	4.8	0.8
	QAMS		60.2		12.9		6.7		13.6		4.9	
